# Improving Consent Documentation in the Medical Intensive Care Unit

**DOI:** 10.7759/cureus.6174

**Published:** 2019-11-17

**Authors:** Armin Krvavac, Pujan H Patel, Ghassan Kamel, Zeyu Hu, Nirav Patel

**Affiliations:** 1 Pulmonary & Critical Care, University of Missouri Healthcare, Columbia, USA; 2 Respiratory Medicine, Royal Brompton Hospital, London, GBR; 3 Internal Medicine - Critical Care, Saint Louis University School of Medicine, St. Louis, USA; 4 Medicine, Louisiana Children's Medical Center (LCMC) Healthcare, New Orleans, USA

**Keywords:** quality improvement, compliance, critical care, informed patient, informed consent, educated patient, shared decision-making

## Abstract

The contemporary patient-centered medical practice relies upon the acquisition of informed consent, which serves as written proof that the patient has recognized and agreed to the risks and benefits of their treatment. Well-documented informed consent forms are not only reflective of important ethical practices in medicine but can also serve as legal documents to protect healthcare providers from undue liabilities. We conducted a quality improvement project with the intention to improve the accuracy and completeness of consent form documentation in the medical intensive care unit.

The evaluation of consent forms before our intervention revealed that only 6.8% were correctly completed, with an average of 10.2 out of 14 (73%) essential items correct. Our intervention involved a multifaceted approach that included targeted education in combination with process improvement. The post-intervention results at one month revealed improvement in consent form accuracy from 6.8% to 60% (p = 0.0001), with an increase in the average number of essential items documented correctly from 10.2 to 13.5 (p = 0.0001). Data were collected three months post-intervention to evaluate for sustained improvement. Results revealed a significant decrease in consent form accuracy to 39% when compared to the one-month post-intervention data but still maintained a statistically significant improvement when compared to initial baseline data; 6.8% to 39% (p = <0.01).

Following the intervention, overall consent form accuracy improved significantly at our institution. Furthermore, these positive adjustments persisted when assessed at three months post-intervention despite the decrease as compared to one-month post-intervention. This trend suggests that our multifaceted intervention was able to increase the quality and accuracy of consent form documentation successfully.

## Introduction

Over the past few decades, the practice of medicine has transitioned to placing a greater emphasis on shared physician-patient decision-making. This transformation has not only been reflected in the Institute of Medicine’s recommendations of physician core competencies for delivering safer, more efficient, evidenced-based care but also in the results of numerous studies that found the recognition of patient preferences to be correlated with better health outcomes and higher patient satisfaction ratings [1-3].

A foundational component that contemporary patient-centered medical practice relies upon is the acquisition of informed consent. This serves as documentary proof that the patient has recognized and agreed to the risks and benefits of their treatment. This is especially important in intensive care units (ICUs) where a great number of critical yet invasive interventions are frequently performed [4]. Additionally, the Code of Federal Regulations 45 CFR § 164.530(j)(2) also requires that patient records be maintained for at least six years from the date of when they were last in effect. Taken together, well-documented consent forms are not only reflective of important ethical practices in medicine but also serve as legal documents to protect healthcare providers from undue liabilities [5]. Nevertheless, medical audits have continued to show inadequate documentation regarding informed decision-making for decades [6-8].

Regulatory surveys revealed inconsistent completion of the informed consent documentation at our institution. In response, we developed a quality improvement project with an aim to improve the accuracy and completeness of consent form documentation in the medical intensive care unit (MICU). In this study, we report positive changes in the quality of informed consent documents in response to educational interventions such as short teaching sessions, reminder posters, and pocket cards.

## Materials and methods

Consent process

The SSM Health Saint Louis University Hospital is a tertiary care, Level I trauma academic center with a total of 356 beds, located in Saint Louis, Missouri. The MICU service is composed of three teams; each with one attending physician, one subspecialty fellow, and a varying number of residents and interns. A night float team, consisting of a subspecialty fellow, nurse practitioner, resident, and intern, covers all three teams during the night. Subspecialty fellows, residents, and interns rotate on the service for a month at a time while attending physicians rotate on for one to two weeks at any given time. Procedures are performed by all members of the care team mentioned and consents are, therefore, also obtained by these members of the care team.

Consents are obtained directly from the patient by the physician or nurse practitioner when patients are alert and deemed competent to provide consent. Surrogate decision-makers or designated durable power of attorney for healthcare decisions are used when a patient’s clinical status impairs his/her ability to give informed consent. The physician or nurse practitioner obtaining consent informs the patient or surrogate decision-maker of the type of procedure being performed, the reason for the procedure, the risks of the procedure, the benefits of the procedure, alternatives to the procedure, and the need for sedation or transfusion with the procedure. Once informed consent for the procedure is given, the physician or nurse practitioner complete the consent form. Next, the patient or surrogate decision-maker and physician sign and date the consent form. The consent form is then reviewed and witnessed by another member of the care team, most typically the patient’s nurse.

**Figure 1:**
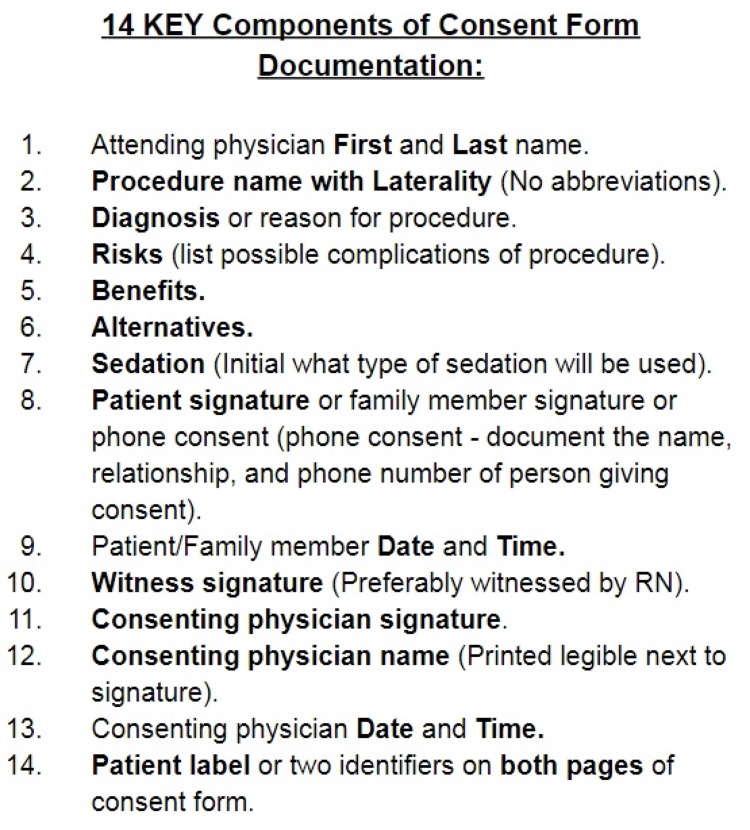
Essential Components of Consent Form: Pocket Card

We identified 14 essential components on the consent form in line with the hospital consent policy as listed in Figure 1.

Pre-intervention data collection

All patients primarily managed by our facility’s medical ICU team had their physical besides charts audited for the presence of procedural consent forms. The pre-intervention data were collected by reviewing consent forms completed by the three MICU teams from November 7, 2017, through November 14, 2017. Data obtained included all 14 of the key components listed in Figure 1. Additionally, we recorded which physician (attending, fellow, or resident) obtained the consent and which MICU team obtained the consent.

Metrics

We identified 14 essential components that should be included in the consent form that is in line with the current consent policy at our institution. Each form was binarily scored based on the accurate completion of each of the 14 required components of the consent form. A total score out of 14 was given to every consent form.

Intervention

A pre-intervention data analysis was performed and multiple factors contributing to a decrease in consent form accuracy were identified. Lack of physician name, diagnosis, benefits, and alternatives were identified as the most frequently missed components. Subsequently, a series of interventions were introduced by our team and organized on an effort-yield table to identify high yield-low effort options that would provide the biggest improvement.

Our first intervention focused on raising awareness about the strikingly low accuracy of consent form documentation among residents, fellows, and attendings on the MICU teams. We presented the pre-intervention data to the division of pulmonary, critical care, and sleep medicine faculty and fellows. Next, a short teaching session detailing all 14 essential components of the consent form was given to all the members of each MICU team. Signs detailing the importance of the consent process and accurate consent documentation were posted in the resident and fellow workrooms. Additionally, laminated reminder pocket cards were provided to the attendings, fellows, and residents (Figure 1).

The second part of our intervention focused on process improvement. For this, we sought buy-in from the intensive care unit nursing staff. Specifically, we educated nursing leaders and nursing staff about two process improvements. First, the nursing staff was instructed to verify the accuracy of the consent after witnessing the consent process. Second, the nursing staff was instructed to again verify the accuracy of the consent along with the rest of the team during the “pre-procedure time-out” process, which is mandated at our institution prior to the start of any procedure. Staff was asked to delay the procedure and rectify the consent before proceeding if there were errors or omissions noted on consent forms. This added two additional checkpoints, including a hard stop, to ensure the accurate completion of the consent form.

Post-intervention data collection

The same data parameters collected prior to the intervention were again collected for all patients primarily managed by our facility’s MICU teams from December 12, 2017, to December 19, 2017 (one-month post-intervention) and February 13, 2018, to February 20, 2018 (three months post-intervention). A post-intervention data analysis was performed and compared to the pre-intervention results using Fisher's exact test for statistical analysis.

## Results

Pre-intervention

The baseline data were collected from November 7, 2017, through November 14, 2017. During this time frame, a total of 73 procedures requiring consent were performed by the three MICU teams. There were only five out of 73 (6.8%) consents in the pre-intervention group that had all 14 of the essential components documented correctly. The average number of accurate components was 10.2 out of 14 (73%) in the pre-intervention group. Evaluation of each component was done so that education could be focused on certain areas. The scores for each essential component is noted in Table 1. Components that scored less than 75% correct include the full name of the physician performing the procedure, diagnosis or reason for the procedure, benefits of the procedure, alternatives to the procedure, and consenting physician name. Additionally, the witness signature whose omission was cited during a recent regulatory survey was correct on only 86% of the consent forms.

**Table 1:** Results: Scores Based on Each Essential Component

	Pre-Intervention	One Month Post-Intervention	Three Months Post-Intervention		p-values	
	% Correct (n=73)	% Correct (n=53)	% Correct (n=77)	Pre vs One Month	Pre vs Three Month	One vs Three Month
Physician Name	31.5%	94.3%	79.2%	<0.01	<0.01	0.02
Procedure Name	100.0%	98.1%	98.7%	0.42	1	1
Diagnosis	67.1%	88.7%	90.9%	<0.01	<0.01	0.77
Risk	95.9%	100.0%	100.0%	0.26	0.11	1
Benefits	41.1%	98.1%	84.4%	<0.01	<0.01	0.01
Alternatives	24.7%	98.1%	81.8%	<0.01	<0.01	<0.01
Sedation	87.7%	96.2%	94.8%	0.12	0.15	1
Patient Signature	90.4%	100.0%	97.4%	0.02	0.09	0.51
Patient Date/Time	84.9%	96.2%	100.0%	0.72	<0.01	0.16
Witness Signature	86.3%	100.0%	85.7%	<0.01	1	<0.01
Consenting Physician Signature	98.6%	100.0%	100.0%	1	0.49	1
Consenting Physician Name	35.6%	92.5%	77.9%	<0.01	<0.01	0.03
Physician Date/Time	94.5%	96.2%	100.0%	1	0.05	0.16
Patient Label Both Pages	83.6%	88.7%	94.8%	0.45	0.03	0.32
Average Score	10.2	13.5	12.9	<0.01	<0.01	0.2
% Perfect Score (14/14)	6.8%	60.4%	39.0%	<0.01	<0.01	0.02

Post-intervention

Post-intervention data were collected at one- and three- month intervals following the interventions. This was done to evaluate the sustained effect of interventions over time. The one-month post-intervention data showed marked improvement in consent form accuracy. Data evaluating 53 consent forms were collected between December 12, 2017, to December 19, 2017, and revealed a statistically significant improvement in the number of consents with all 14 essential components documented correctly; 6.8% (5/73) to 60.4% (32/53), p = <0.01. Additionally, the average number of essential items documented correctly improved significantly from 10.2 to 13.5 out of 14 (p = <0.01). Statistically significant improvements were noted in the proper documentation of physician name, benefits, alternatives, patient signature, and witness signature. Detailed scores for each subcomponent along with p-alues comparing data to pre-intervention and one-month post-intervention are shown in Table 1.

**Figure 2:**
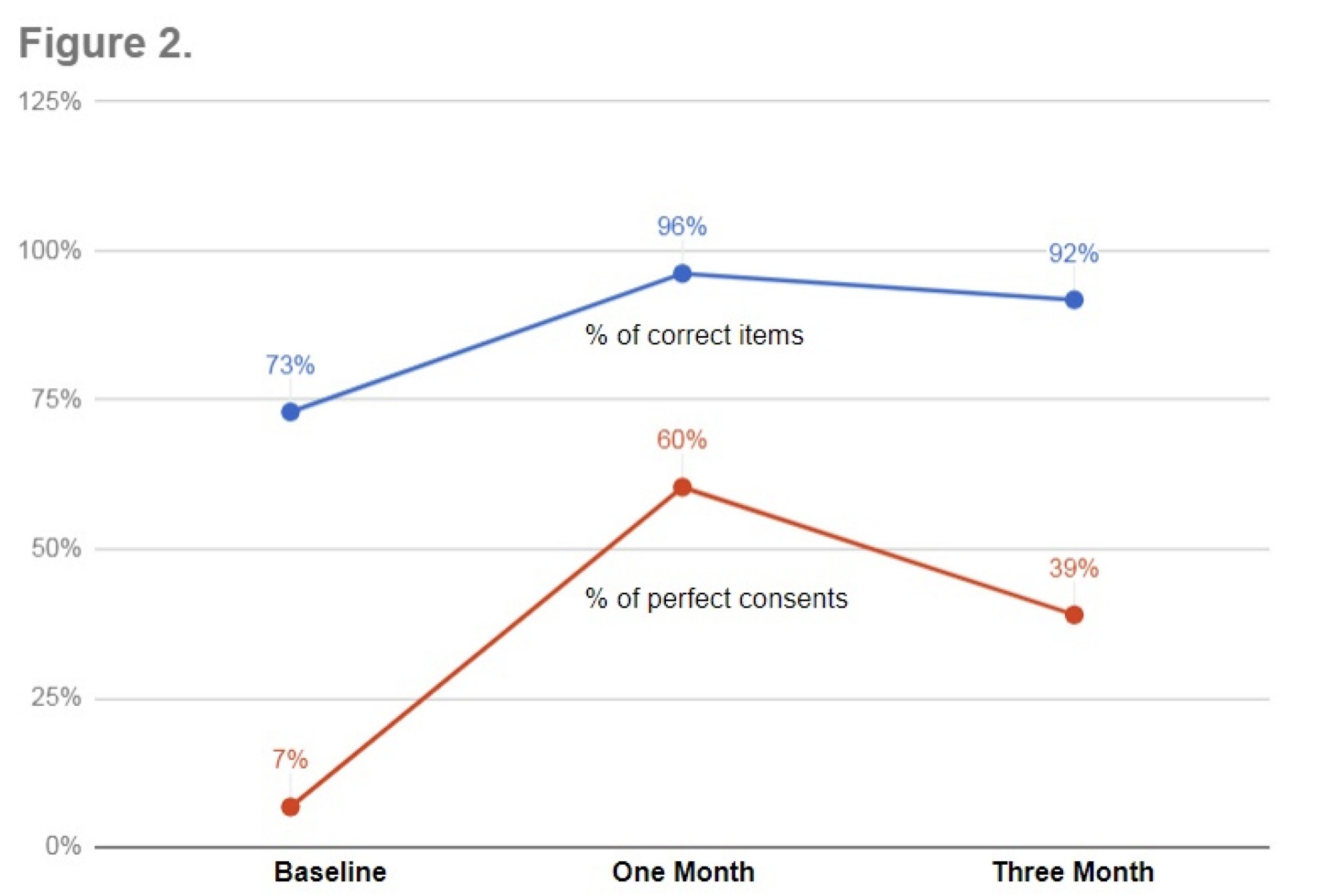
Results: Change in Consent Form Accuracy over Time

The three-month post-intervention data evaluating the sustained effect of interventions were collected from February 13, 2018, to February 20, 2018. Data from a total of 77 consent forms were collected during this period. The number of consents with all 14 essential components documented correctly decreased to 39% (30/77) when compared to one-month post-intervention data. However, it still maintained a statistically significant improvement when compared to baseline data; 6.8% to 39% (p = <0.01). The average score did not decrease significantly when compared to one-month post-intervention (13.5 to 12.9 (p = 0.2)) but did maintain the statistically significant improvement compared to baseline data (p = <0.01) (Figure 2).

## Discussion

Informed consent forms serve as documented evidence of patients’ acknowledgment to proceed with their care upon competent assessments of the associated risks and benefits [5]. Therefore, it is important for the forms to be completed correctly during the course of the patients’ hospitalization. Prior groups have demonstrated improvement in the consent form process in the intensive care setting with simple interventions [8-10]. The objective of our study was to improve the accuracy of informed consent documentation in the MICU. Prior to any intervention, we were surprised to find that many of the seemingly intuitive consent form components, such as “physician name,” were correctly completed less than a third of the time, and even patient diagnosis, arguably one of the most important components, had only a 67.1% correct completion rate. Some of the poor accuracies could have been attributable to the inherent design flaws of the consent form itself. For example, the physician name section never explicitly indicated that both first and last names must be written. However other components, such as the "benefits" and "alternatives" sections, despite being explicitly indicated on the consent form, also received <50% correct rates, suggesting there was an underlying inattention when clinicians are filling out these forms.

Following our intervention, we were able to demonstrate that all five categories that received a <75% correct rating in the pre-interventional stages (physician name, diagnosis, benefits, alternatives, and consenting physician name) demonstrated statistically significant improvements at one-month (p < 0.01) post-intervention. Furthermore, these positive adjustments persisted three months post-intervention as compared to pre-intervention data (p < 0.01). This trend suggests that our multifaceted approach to education and raising awareness was able to successfully increase the quality of consent form documentation at least in the short term. However, while most consent form components maintained their standards after three months, we identified five components (physician name, benefits, alternatives, witness signature, consenting physician name) that demonstrated a statistically significant decline in percent correct when evaluated at three months post-intervention compared to one month post-intervention (p < 0.01). These decreases likely suggest time-dependent regressions that can be improved through further reinforcement by means of repeated training sessions with incoming staff, reminders, or other more iterative interventions such as re-engineering the consent form and enhancing the time-out process.

Specifically, redesigning the consent form such that the requirements for each component are clearly and intuitively outlined will likely help with compliance. Attention will be made to ensure that the new consent form maintains all the required elements to reduce therapeutic misconceptions and a lack of patient understanding. Prior data has demonstrated that many consent forms do not contain all of the required elements to facilitate patient understanding and retention [10-11]. Additionally, the development of procedure-specific labels and consent forms will be considered, as they have previously demonstrated improvement in consent form accuracy [12]. Furthermore, the implementation of electronic and interactive versions of these consent forms would improve both the accuracy of documentation and patient understanding/informed decision-making [13]. In doing so, we hope to minimize previously mentioned time-dependent regressions and consequently improve the overall standards at which consent forms are documented.

Moreover, we believe that enforcing the proper documentation of consent forms should not be exclusively the clinicians’ responsibility but rather a joint effort by all members of the multidisciplinary care team, which includes nurses, pharmacists, and patient care technicians. To this extent, we will be considering incorporating a brief, formal presentation on how to properly document consent forms during orientation training for all new staff members working at our institution. We anticipate favorable outcomes upon making these changes.

Our study has several limitations. First, it’s performed at one medical center and only in the medical intensive care unit. However, our intervention achieved successful results that can now be expanded hospital-wide. Second, our study did not address the issues of the consent form format, which could be contributing to improper consent form documentation. This will be addressed with hospital administration as part of the next phase of our interventions. Third, our intervention did not include education on procedure-specific consent form documentation but this can be considered in future projects when this is applied hospital-wide.

Informed consent will continue to play an important role in the ethical and practical care of patients. The 1994 ruling (Butler v. South Fulton Medical center) stated that “even if the patient is provided proper and legal disclosure, he or she must comprehend what the physician is saying and understand the information on the consent form so that he or she gives permission for treatment or surgery voluntarily” [14-15]. Unfortunately, the average consent form and consent form process generally do not achieve this goal of substantial understanding [16-17]. Therefore, our team recognized the importance of focusing future research and investigative interventions on not only improving the accuracy of consent form documentation but also focusing on improving information delivery to the patient to truly enhance the informed consent process. A review of 65 randomized controlled trials involving patients undergoing a variety of procedures demonstrated various effective strategies that significantly improve patient knowledge and understanding during the consent process [18]. Hospital-wide employment of similar measures at our facility could further enhance and improve the practicality of the consent form process.

## Conclusions

The practice of medicine has transitioned to placing a greater emphasis on shared physician-patient decision-making. The acquisition of informed consent is a foundational component of this shared decision-making process that also serves as documentary proof that the patient has recognized and agreed to the risks and benefits of their treatment. Despite its importance, medical audits have continued to show inadequate documentation regarding informed decision-making for decades. Furthermore, the consent form and consenting process generally do not achieve true patient comprehension. In this study, we report positive changes in the quality of informed consent documents in response to educational interventions such as short teaching sessions, reminder posters, and pocket cards. Our team recognizes the importance of focusing future research and investigative interventions on not only improving the accuracy of consent form documentation but also focusing on improving information delivery to the patient to truly enhance the informed consent process. No single intervention will be sufficient. Various effective strategies that significantly improve consent form documentation, the consenting process, and patient knowledge and understanding need to be employed on a system-wide basis.
